# Influence of neighbourhood purchasing power on breastfeeding at four months of age: a Swedish population-based cohort study

**DOI:** 10.1186/1471-2458-13-1077

**Published:** 2013-11-15

**Authors:** Gerd Almquist-Tangen, Ulf Strömberg, Anders Holmén, Bernt Alm, Josefine Roswall, Stefan Bergman, Jovanna Dahlgren

**Affiliations:** 1 Child Health Care Unit, Region Halland SE-434 80, Kungsbacka, Sweden; 2 Research and Development Department, Halland, Sweden; 3 Department of Paediatrics, Halland Hospital, Halmstad, Sweden; 4 Research and Development Centre, Spenshult Hospital, Oskarström, Sweden; 5 Department of Paediatrics, Institute for Clinical Sciences at the Sahlgrenska Academy, University of Gothenburg, Gothenburg, Sweden

**Keywords:** Breastfeeding, Child health, Neighbourhood purchasing power, Prevention, Spatial determinant

## Abstract

**Background:**

Parental socioeconomic status (SES) is an important determinant in child health, influencing beneficial factors such as breastfeeding. A better understanding of the influence of neighbourhood-level SES measures, relating to spatial determinants, might lead to targeted actions to promote breastfeeding during infancy.

**Methods:**

A cross-sectional study analysis the association between breastfeeding at four months of age and neighbourhood purchasing power, taking account of individual-level variables including maternal age, smoking and parental level of education. Data were obtained from a prospective population- based cohort study recruited from birth in 2007–2008 in the Halland region, southwestern Sweden. Questionnaire data on the individual-level variables and the outcome variable of breastfeeding at four months (yes/no) were used (n = 2 407). Each mother was geo-coded with respect to her residential parish (there are 61 parishes in the region) and then stratified by parish-level household purchasing power. It emerged that four neighbourhood characteristics were reasonable to use, *viz*. <10%, 10-19%, 20-29% and ≥ 30% of the resident families with low purchasing power.

**Results:**

The proportion of mothers not breastfeeding at four months of age showed a highly significant trend across the neighbourhood strata (p = 0.00004): from 16.3% (< 10% with low purchasing power) to 29.4% (≥ 30% with low purchasing power), yielding an OR of 2.24 (95% confidence interval: 1.45-3.16). After adjusting for the individual-level variables, the corresponding OR = 1.63 (1.07-2.56) was significant and the trend across the strata was still evident (p = 0.05). A multi-level analysis estimated that, in the neighbourhoods with ≥ 30% of the families with low purchasing power, 20% more mothers than expected, taking account of the individual-level factors, reported no breastfeeding at four months of age (≥ 95% posterior probability of an elevated observed-to-expected ratio).

**Conclusion:**

The neighbourhood purchasing power provided a spatial determinant of low numbers of mothers breastfeeding at four months of age, which could be relevant to consider for targeted actions. The elevated observed-to-expected ratio in the neighbourhoods with the lowest purchasing power points toward a possible contextual influence.

## Background

Human breast milk is considered to be the best nutrition for newborns and infants, as it contains the optimal ingredients for healthy growth and development [1]. In May 2001, the World Health Assembly (WHA) passed Resolution 54.2, stating that the optimal length for exclusive breastfeeding is six months and that breastfeeding should continue into the second year of life [2]. Although 97% of Swedish mothers start initiate breastfeeding, little more than half (52%) are breastfeeding by six months [3]. The scientific literature regarding factors associated with breastfeeding duration is extensive i.e. sociodemographic, biomedical and psychosocial determinants and health- care organisation are important and interact with one another [4-6].

It has been hypothesized that neighbourhood influences affect individual health behaviour through a variety of mechanisms, including the availability of health care, community norms and values, isolation from people with different healthy behaviour, access to health promotion messages and psychological stress associated with living in a disadvantaged neighbourhood [5-8]. Breastfeeding and a high SES have consistently been found to correlate in such a way that highly educated, non-smoking, privileged mothers are more likely to initiate and continue breastfeeding for a longer period of time [9-12]. Smoking, early breastfeeding cessation and other similar habits could reflect a higher social acceptance of inappropriate behaviours in a suboptimal community setting [13]. By examining patterns of association for different SES indicators, an additional understanding could be acquired by considering neighbourhood-level characteristics that could identify and then target certain areas with an evidently unfavourable outcome. Parental socioeconomic status (SES) is an important determinant in child health, influencing beneficial factors such as breastfeeding. For example, the geo-mapping of the childhood caries risk has prompted targeted preventive programmes [14]. By providing evidence of spatial determinants of breastfeeding during infancy, decision makers can be helped to identify relevant criteria to set priorities in their specific areas. In this way, a better understanding of the influence of neighbourhood SES measures can lead to targeted actions for promoting breastfeeding during infancy.

We showed in a previous study that most mothers (91.9%) were breastfeeding at one month of age. A correlation was found between low parental education and smoking, low gestational age, low birth weight, pacifier use and breastfeeding difficulties and early breastfeeding discontinuation [15]. In the follow-up study, many mothers (58.3%) were breastfeeding at six months of age [16].

The aim of the current study was to evaluate possible association between low numbers of mothers breastfeeding at four months of age and neighbourhood purchasing power, taking account of individual-level variables including maternal age and smoking, and parental level of education.

## Methods

This cross-sectional study is part of a larger Swedish project called the “Halland Health and Growth Study (H^2^GS)”. The main goal of the H^2^GS is to increase our understanding of the concept of child health and growth from a parental perspective, focusing on parental needs, and a medical/social perspective, elucidating risk factors for growth disturbances. A more specific aim within the project, which is expressed in this study, is to explore the effect of neighbourhood purchasing power, maternal age and smoking, and parental educational level on low numbers of mothers breastfeeding at four months of age.

The H^2^GS is a prospective, longitudinal-, population-based birth cohort study that recruited children born in Halland, south-western Sweden, between 1 October 2007 and 31 December 2008. The study protocol, recruitment strategy and the representativeness of the sample have been reported elsewhere [15].

### Response rate

The children were followed-up at one, four, six, 12 and 18 months and at two, three, four and five years of age, but only data relating to four months of age were used in this study. In all, there were 3,860 births in Halland during the recruitment period. Of these, the parents of 2,666 infants chose to participate, which gave a response rate of 69.2%. However, 376 parents actively chose not to participate (9.7%) and 814 did not respond (21.1%). At four months of age, the response rate was 2,544 (65.9%).

### Individual level

Information on maternal smoking (non-smoker, light smoker or heavy smoker) and parental educational attainment; low (did not complete high school), medium (high school) or high (university or tertiary qualification) was self-reported. In the analyses, we included n = 2, 407 families with complete data for the individual-level variables. Maternal age ranged between 15–46 years, 5.5% of the mothers were smokers and 5.3% did not complete high school.

### Neighbourhood socio-economy

**Figure 1:**
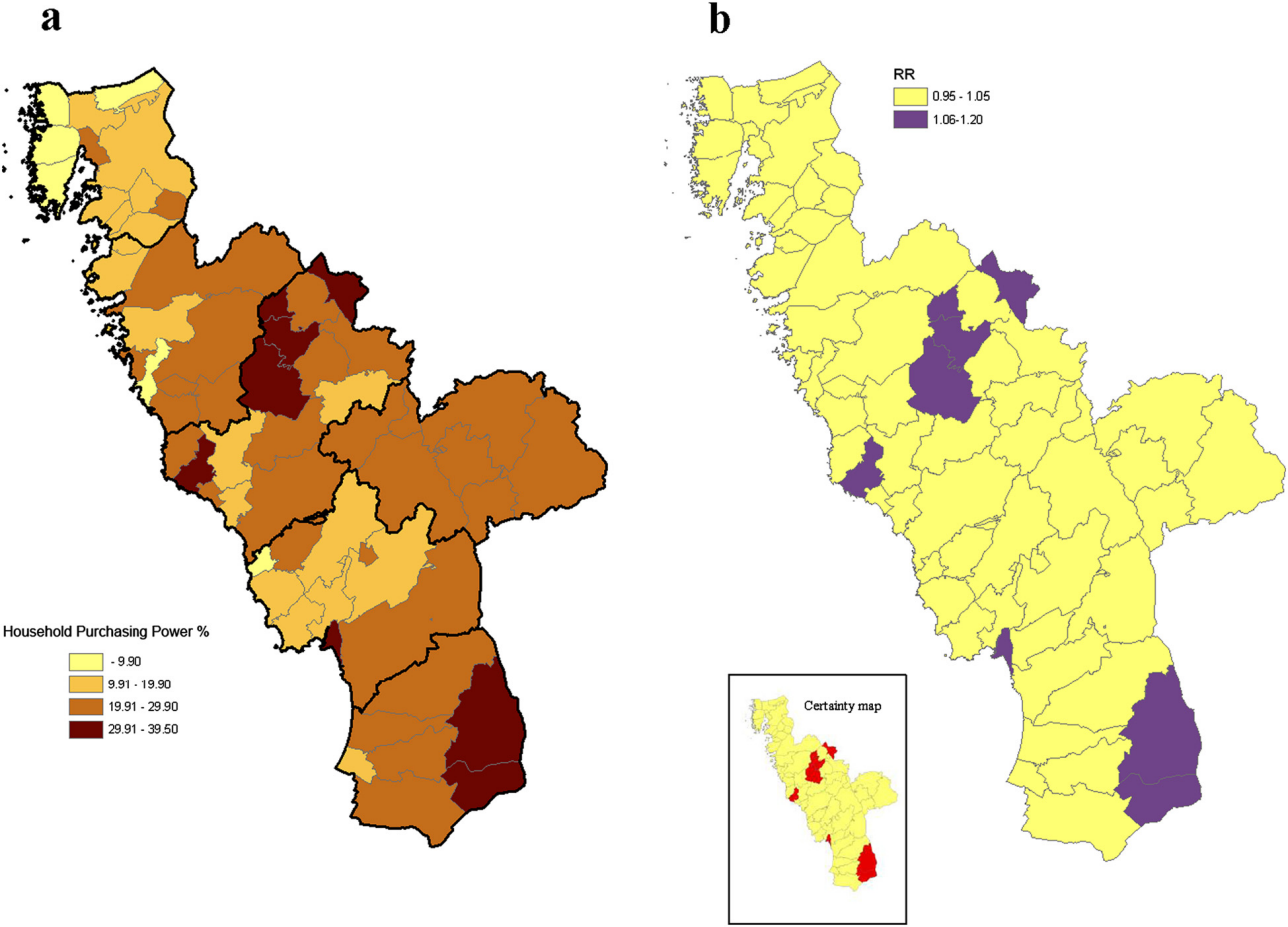
**Geo-map of neighbourhood household purchasing power.** The residential areas (parishes) were classified into < 10%, 10–19%, 20–29% and ≥ 30% based on this indicator [according to the Swedish standard, corresponding to ≤ USD 19,500 annual household purchasing power among all resident families with at least one child (≤ 19 years old: family with the same residential address)]. Neighbourhood household purchasing power was defined as total family disposable income adjusted for the composition of the family (number of adults and children). **(b)** The corresponding geo-map, based on the grouping of the parishes according to neighbourhood-level purchasing power **(a)**, for relative risks of discontinuing breast-feeding at four months of age, denoted RR*i* for group *i*, with adjustments for the individual-level variables of maternal age, smoking and parental level of education. Moreover, the accompanying statistical certainty map is shown; neighbourhood household purchasing power areas with a posterior probability of an elevated RR*i* [Pr (RR*i* > 1|data)] above 95% coloured in red.

Each mother was geo-coded with respect to her residential parish (there are 61 parishes in the region). Statistics Sweden provided parish-level data from 2010 relating to the socio-economic indicator we were considering, *viz*. the proportion of families with low household purchasing power (according to the Swedish standard, corresponding to ≤ USD 19, 500 annually household purchasing power) among all resident families with at least one child (≤ 19 years old: family with the same residential address). Neighbourhood purchasing power was defined as total family disposable income adjusted for the composition of the family (number of adults and children). The parishes were classified into < 10%, 10-19%, 20-29% and ≥30%, based on this indicator (Figure 1a). The four neighbourhood strata were chosen based on the statistical analyses.

## Ethics

The study was approved by the Research Ethics Committee at Lund University (study number 299/2007). Written consent was obtained from the parents involved.

## Statistical methods

The primary outcome variable was breastfeeding at four months of age (yes/no). In the crude analysis, outcome data were compared across the strata by neighbourhood purchasing power using the Cochran-Armitage test for trends. Crude odds ratios (cORs) with 95% confidence intervals (CI’s) were also estimated. The chosen neighbourhood strata reasonably revealed the trend in the crude OR. Consideration of finer stratification implied similar crude ORs in adjacent strata: these strata were therefore collapsed. A multivariate logistic regression analysis was then performed, taking account of the confounding effects of individual-level variables. Adjusted odds ratios (aORs) with 95% CIs were thereby estimated, reflecting the effects of each explanatory variable.

Multi-level modelling distinguishes individual and neighbourhood levels of information in a model [17]. Moreover, we performed a multi-level analysis in order to estimate the effect of neighbourhood purchasing power in another way. This estimation was performed by employing a hierarchical Bayesian model, using a prior Gamma-model for the neighbourhood-level relative risks of discontinuing of breast-feeding at four months of age, denoted RR, for stratum *i*[18]. More specifically, we first calculated the observed and expected numbers of mothers not breast-feeding at four months of age, denoted O_*i*_, and E_*i*_ respectively, for neighbourhood stratum *i*. The expected numbers were calculated using the crude and the adjusted approach, i.e. without and with additional stratification by the individual-level variables. An estimation of the RR_*i*_ for neighbourhood stratum *i* was then carried out by applying the conventional statistical Poission model to the observed numbers [O_*i*_ ~ Poisson (RR_*i*_ × E_*i*_)] with empirical Bayes smoothing of the RR_i_:s across the neighbourhood strata using a prior Gamma-model [RR_*i*_ ~ Gamma(α, β)]. Bayesian smoothing of this kind yielded “shrinkage” of the conventional observed-to-expected ratios towards the expected average (i.e., O/E = 1), which can be justified statistically [17]. We also calculated the posterior probability of an elevated RR_*i*_ [Pr (RR_*i*_ > 1|data)].

IBM SPSS 20.0.2 and StatXact 6.2.0 (Cytel Inc., Cambridge, MA, USA) were used for the conventional statistical analyses. The multi-level analysis was performed using the Rapid Inquiry Facility free software [19].

## Results

A total of 1, 898 (78.7%) infants were breastfed at four months of age, which does not differ significantly notably from the official Swedish breastfeeding statistic of (79.6% in 2007) [3]. The lower-middle purchasing power class neighbourhood had the highest proportion of maternal smoking (8.7%) and young mothers, defined as < 20 yrs (2.7%) (Table 1). Parental educational level showed a descending trend from the highest to the lowest purchasing power class neighbourhood.

**Table 1:** Descriptive characteristics of the study population based on their neighbourhood-level (parish) purchasing power

	n^*^	**Not breast-feeding at 4 m (%)**	**Mother ≤20 yrs (%)**	**Mother smoker (%)**	**Mother and father without/with post-secondary education (%)**
*Neighbourhood purchasing power*^†^					
<10	374	16.3	0.8	0.8	20.4/47.4
10–19.9	689	19.2	0.3	2.5	27.9/37.8
20–29.9	1106	22.7	2.7	8.7	45.0/25.8
30+	238	29.4	0.9	7.2	46.8/22.1

^*^Number of mothers with data on breast-feeding at four months. There are additional missing data on maternal age (n = 17), maternal smoking (n = 21) and parental educational level (n = 160).

^†^Proportion (%) of families with low neighbourhood household purchasing power (according to Swedish standards; < USD 19 500 household purchasing power) among all resident families with at least one child (up to 19 years old) in a neighbourhood area (parish). Neighbourhood household purchasing power was defined as total family disposable income adjusted for the composition of the family (number of adults and children).

The proportion of mothers not breastfeeding at four months of age showed a highly significant trend across the neighbourhood strata (p = 0.00004): from 16.3% (<10% with low purchasing power) to 29.4% (≥ 30% with low purchasing power), yielding an odds ratio (cOR) of 2.24 (95% confidence interval: 1.45-3.16). After checking for the individual-level variables, the corresponding aOR of 1.63 (1.07-2.56) when comparing the lowest with the highest stratum and the trend across the strata was almost significant (p = 0.052) (Table 2). As expected, the individual-level variables had a pronounced effect.

**Table 2:** Association between breastfeeding at four months of age and neighbourhood household purchasing power, without and with adjustments for individual-level variables

***Explanatory variable***	**Neighbourhood-level without adjustments**	**Neighbourhood-level with adjustments**
**Category**	**OR (95% CI)**	**OR (95% CI)**
Neighbourhood purchasing power^*^		
< 10	1.00 (reference)	1.00 (reference)
10–19.9	1.21 (0.87-1.70)	1.05 (0.74-1.48)
20–29.9	1.51 (1.11-2.05)	1.09 (0.79-1.52)
30+	2.14 (1.45-3.16)	1.63 (1.07-2.46)
*p-value for trend*^†^	*0.00004*	*0.052*
*Maternal age (yrs)*	- ^‡^	
≤ 20		2.49 (1.24-4.99)
21+		1.00 (reference)
*Maternal smoking*	- ^‡^	
No		1.00 (reference)
Yes		2.57 (1.74-3.80)
*Maternal and paternal education*	- ^‡^	
Both post-secondary		1.00 (reference)
One post-secondary		1.44 (1.09-1.92)
None post-secondary		2.07 (1.58-2.73)

^*^Proportion (%) of families with low neighbourhood household purchasing power (according to Swedish standards; < USD 19 500 neighbourhood household purchasing power) among all resident families with at least one child (up to 19 years old) in a neighbourhood area (parish). Neighbourhood household purchasing power was defined as total family disposable income adjusted for the composition of the family (number of adults and children).

^†^Cochran-Armitage trend test (two-sided p-value).

^‡^Not included.

The multi-level analysis revealed that, in the neighbourhoods with ≥ 30% of the families having low purchasing power, 20% more mothers than expected taking account of the individual-level factors, reported no breastfeeding at four months of age (≥ 95% posterior probability of an elevated RR) (Table 3, Figure 1b).

**Table 3:** Association between breastfeeding at four months of age and neighbourhood purchasing power, without and with adjustments for individual-level variables

	**Neighbourhood-level without adjustments**				Neighbourhood-level with adjustments^*^			
	**Obs**	**Exp**	**O/E (95% CI)**^**†**^	**RR**^**‡**^	**Obs**	**Exp**	**O/E (95% CI)**^**†**^	**RR**^**‡**^
*Neighbourhood purchasing power*^§^								
<10	61	79.9	0.76 (0.58-0.98)	0.80	60	54.6	0.93 (0.71-1.20)	0.97
10–19.9	132	147.1	0.90 (0.76-1.06)	0.91	117	123.3	0.95 (0.79-1.14)	0.97
20–29.9	251	236.2	1.06 (0.94-1.20)	1.06	229	233.5	0.98 (0.86-1.12)	0.99
30+	70	50.8	1.38 (1.07-1.74)	1.30**	65	49.7	1.31 (1.01-1.67)	1.20**

Results expressed as observed (Obs) and expected (Exp) numbers of mothers not breastfeeding at four months of age, obtained from multi-level analyses, without and with adjustment for individual-level variables.

^*^By stratification of maternal age (≤20, 21+), maternal smoking (no, yes) and parental education (both parents with post-secondary education;

one parent with this educational level; or none).

^†^Obs/Exp, with 95% confidence interval.

^‡^Relative risk of discontinuing of breast-feeding at four months of age estimated from a multi-level analysis; **indicates an elevated RR with posterior probability, Pr (RR > 1│data), of >95%.

^§^Proportion (%) of families with low neighbourhood household purchasing power (according to Swedish standards; < USD 19 500 household purchasing power) among all resident families with at least one child (up to 19 years old) in a neighbourhood area (parish). Neighbourhood household purchasing power was defined as total family disposable income adjusted for the composition of the family (number of adults and children).

## Discussion

Neighbourhood data, reflecting contextual SES, were available at parish-level. It could not be assumed that each parish was a homogeneous spatial area in terms of SES. Nevertheless, it emerged that the method, when applied to categorising neighbourhood purchasing power, based on parish-level data, revealed a contextual effect. It is possible that, other spatial areas might have revealed a more pronounced contextual effect.

We considered neighbourhood purchasing power as the primary indicator of neighbourhood socio-economy. This indicator takes only resident families with at least one child (≤ 19 years of age) into account: the elderly population was ignored, which can be justified. In this study, low neighbourhood purchasing power was shown to be significantly associated with the risk of not breastfeeding at four months. The reason for choosing breastfeeding at four months is the WHO recommendation and, as a result, a measurement of compliance with optimal current infant feeding beliefs. The findings are consistent with a growing body of literature suggesting that the SES and neighbourhood areas have an effect on lifestyle behaviour such as breastfeeding [10,19,20]. Sweden ranks among the top countries in the Organisation of Economic Cooperation and Development (OECD) Better Life Index [21], with a high standard of living, well-educated population offering parents a well-developed parental insurance and parental leave programme. This study demonstrates that, despite these efforts, there are substantial SES differences and, for this reason, an updated report on health inequalities in Sweden has been produced.

The new finding in the present study is that neighbourhood purchasing power is still a determinant of breastfeeding when maternal age, smoking and parental education are adjusted for. We have shown substantial differences in maternal age, smoking and parental education across the neighbourhood household purchasing power strata. An expected gradient was observed between parental education and neighbourhood household purchasing power, i.e. families with lower neighbourhood purchasing power were associated with lower educational attainment. These findings are consistent with previous research documenting the SES gradient and educational attainment [22,23]. Parental education is often presented as a proxy for socioeconomic position; individuals with a higher educational level will most frequently have higher incomes [23]. This demonstrates the need to take account of both parental income and individual characteristics when conducting similar studies. In this study we only had access to aggregated (parish-level) data on neighbourhood purchasing power, *viz*. the proportion of resident families with low household purchasing power. Our objective was to address the influence of neighbourhood purchasing power on breastfeeding at four months of age (with additional interest in the influence of maternal age and smoking and parental educational level). However, it would be of interest to study the influence of household purchasing power.

Lifestyle factors and behaviours that are adopted very early in life tend to persist throughout life [24]. Studies show that investing in quality programmes and services that support the family’s earliest development produces a higher rate of return than investments made later in life [25]. Household characteristics and health-related behaviour are linked with income [23]. However, this relationship is not yet fully understood [22]. Similar approaches have been used but at an individual level, the individual council tax valuation band in the UK (using the estimated value of an individual’s home), for example showed that this index governed maternal beliefs and intentions relating to breastfeeding [20,26].

The elevated risk of low numbers of mothers breastfeeding at four months in the neighbourhoods with the lowest purchasing power, points toward a possible contextual influence, which could be relevant to consider when it comes to targeted actions. Low-income parents who are stretched by a lack of money may have less energy to persevere with breastfeeding or wrestling with children to put on seat belts [22]. Giving birth, breastfeeding and becoming a family occur within a social context and an understanding of this context is essential if health professionals are to work alongside mothers. A woman’s decision to breastfeed or not is influenced by what is socially acceptable, and this decision is open to social and cultural influences [9,27]. Moreover, it appears that parents in the higher SES groups are more likely to have the same opinion and thereby comply with current food and feeding recommendations [9,28].

Most mothers are knowledgeable when it comes to the benefits of breast milk and breastfeeding [9]. However, many studies have shown that the discontinuation of early breastfeeding may be due to several causes, such as breastfeeding difficulties, perceived inconsistent advice and the need to get back to work [15,29,30]. Most attempts to improve breastfeeding rates have focused on mothers and then especially on certain risk groups, *i.e.* young mothers, single mothers and mothers with low educational attainment [27,31]. Breastfeeding interventions that have so far been shown to be the most effective are needs-based, informal repeat educational programmes [12]. However, the key challenge is the recruitment (and retention) of appropriately trained and qualified staff, who are equipped with neighbourhood specific, up-dated and evidence-based material.

The main strength of this study is that it is a large population-based survey, comprising participants from diverse socio-economic and ethnic backgrounds. Another strength is the ability to integrate several explanations in one analysis. The advantage of using neighbourhood purchasing power as a variable is that it takes account of family structure in a residential parish. The socio-economic statistics applied here were from 2010 (when the children were two to three years of age), which could be seen as a weakness, but this was only a minor concern as the neighbourhood characteristics appeared to be stable over the years [14]. The validity of studies showing a correlation between negative effects on children growing up in low SES neighbourhoods i.e. low birth weight, breastfeeding and childhood injury has been questioned because of confounders, reverse causality and individualistic fallacies [13,32]. Nevertheless, the outcome data showed a more evident trend across strata based on neighbourhood purchasing power, as compared with the alternative neighbourhood characteristics.

Given the results, future interventions to promote breastfeeding should adopt a much broader social approach; not only encouraging positive norms for the mother but also engaging the mother’s social network, *i.e.* spouse, grandparents, friends and family, as well as health-care professionals. Furthermore, it is necessary to create breastfeeding friendly premises including the premises at the health care facilities, as well as removing external social barriers to breastfeeding outside the home, offering parental educational programmes and intensive home visiting programmes to mothers who have been assessed as needing additional support.

Policy-makers need to act on inequalities, especially among the child population, which, in the long run, is of economic benefit to society. In this paper we have been able to identify neighbourhood areas in need of expanded support. The challenge lies in offering universal measures, and yet at the same time adapting them, in both scope and design, to those with the greatest needs i.e. proportionate universalism [8]. In order for this to be effective we must make use of this method on a regular basis, monitoring breastfeeding rates, their changes and trends over time in order to address the vulnerable neighbourhood areas at an early stage, as well as monitoring the effect of the intervention programmes. The allocation of preventive resources should be reviewed.

## Conclusion

This study adds further evidence to the notion that privileged mothers living in neighbourhoods with high proportion of families with favourable household purchasing power are less likely to stop breastfeeding before four months of age. On the other hand, there is a greater risk that mothers in low-income neighbourhoods will stop breastfeeding before four months. The neighbourhood purchasing power provided a spatial determinant of low numbers breastfeeding at four months of age, which can be relevant to consider for targeted actions. The elevated observed-to-expected ratio in the neighbourhoods with the lowest purchasing power points toward a possible contextual influence.

## Competing interests

The authors declare that they have no competing interests.

## Authors’ contributions

GAT, JR, BA, SB and JD conceived and designed the study. US and AH conducted the data entry and analysis. All the authors contributed to writing, reviewing and approved the final paper.

## Pre-publication history

The pre-publication history for this paper can be accessed here:

http://www.biomedcentral.com/1471-2458/13/1077/prepub
